# Mammalian Nudt15 hydrolytic and binding activity on methylated guanosine mononucleotides

**DOI:** 10.1007/s00249-023-01678-5

**Published:** 2023-08-29

**Authors:** Maciej Lukaszewicz, Aleksandra Ferenc-Mrozek, Julia Kokosza, Anna Stefaniuk, Janusz Stepinski, Elzbieta Bojarska, Edward Darzynkiewicz

**Affiliations:** 1 Department of Biophysics, Faculty of Physics, University of Warsaw, Pasteura 5, 02-093 Warsaw, Poland; 2 Centre of New Technologies, University of Warsaw, Banacha 2c, 02-097 Warsaw, Poland

**Keywords:** Nudt15, NUDIX family, Methylated mononucleotides, Enzyme kinetics, Differential scanning fluorimetry

## Abstract

The Nudt15 enzyme of the NUDIX protein family is the subject of extensive study due to its action on thiopurine drugs used in the treatment of cancer and inflammatory diseases. In addition to thiopurines, Nudt15 is enzymatically active in vitro on several nucleotide substrates. It has also been suggested that this enzyme may play a role in 5′RNA turnover by hydrolyzing m^7^GDP, a product of mRNA decapping. However, no detailed studies on this substrate with Nudt15 are available. Here, we analyzed the enzymatic activity of Nudt15 with m^7^GDP, its triphosphate form m^7^GTP, and the trimethylated counterparts (m_3_^2,2,7^GDP and m_3_^2,2,7^GTP). Kinetic data revealed a moderate activity of Nudt15 toward these methylated mononucleotides compared to the dGTP substrate. However m^7^GDP and m_3_^2,2,7^GDP showed a distinct stabilization of Nudt15 upon ligand binding, in the same range as dGTP, and thus these two mononucleotides may be used as leading structures in the design of small molecule binders of Nudt15.

**Supplementary Information:**

The online version contains supplementary material available at 10.1007/s00249-023-01678-5.

## Introduction

Nudt15 is a member of the NUDIX (nucleoside diphosphates linked to a moiety x) superfamily of pyrophosphohydrolases that metabolize a broad range of substrates, including (d) NTPs, oxidized nucleotides, capped RNAs, dinucleotide coenzymes (NAD, FAD, dpCoA), and their derivatives (McLennan 2006). NUDIX enzymes are evolutionarily conserved and present in viruses, bacteria, archaea, and eukaryotes. Numerous NUDIX hydrolase genes were identified in mammals (24 in the human genome, McLennan 2006). Their catalytic activity depends on the 23-amino acid Nudix motif, GX_5_EX_7_REUXEEXGU (where U represents a hydrophobic residue and X represents any amino acid), and glutamic acid residues therein (REUXEE) are involved in the binding of divalent metal ions (in most cases Mg^2+^) that are required for the catalytic activity of NUDIX enzymes (McLennan 2006).

Nudt15 was initially proposed to function in the hydrolysis of oxidized nucleotides, similar to another NUDIX enzyme—MTH1—a sanitizer of the oxidized dNTP pool in cells (Gad et.al. 2014; Carter et.al. 2015). However, in contrast to MTH1, Nudt15 showed minimal enzymatic activity toward 8-oxo-dGTP and no activity toward the adenosine nucleotides 2-OH–dATP and 2-OH–ATP (Carter et.al. 2015). siRNA knockdown of Nudt15 in cancer cell lines also had no effect on their survival or increase of 8-oxo-dGTP in DNA, in contrast to siRNA depletion of MTH1 (Carter et.al. 2015). Altogether, these reports indicate that Nudt15 is not primarily involved in the metabolism of oxidized nucleotides.

Independent studies have associated *NUDT15* gene variant (rs116855232, R139C) with intolerance to a thiopurine treatment (such as 6-mercaptopurine, azathiopurine, or 6-thioguanine (Karran and Attard 2008) and with thiopurine-induced adverse drug reactions in patients with acute lymphoblastic leukemia (ALL) and inflammatory bowel disease (IBD) (Yang SK et al. 2014; Yang et al. 2015). Subsequent studies showed the importance of this and several other *NUDT15* variants in thiopurine-induced toxicity (Singh et.al. 2017). Polymorphism in the *NUDT15* gene greatly influences the thiopurine dosage, and ALL patients homozygous in defective *NUDT15* alleles tolerated less than 10% of 6-mercaptopurine dose, in comparison to patients with normal *NUDT15* alleles (Moriyama et.al. 2017). In line with these reports, it was shown that Nudt15 depletion in human colon carcinoma cells increased their sensitivity to 6-thioguanine treatment (Valerie et.al. 2016), and preclinical studies with *NUDT15* knockout mouse model demonstrated that treatment with greatly reduced 6-mercaptopurine dosage maintained anti-leukemic therapeutic efficacy and reduced drug-related toxicity (Nishii et.al. 2018). Since the Nudt15 enzyme appeared to be an important player in the efficacy of thiopurine treatment, specific targeting of Nudt15 with inhibitory compound(s) raised the possibility of improving the efficacy of such treatment in patients with wild-type Nudt15. Recently, examples of such potent and selective Nudt15 competitive inhibitors were developed (Zang et.al. 2020; Rehling et.al. 2021) that were shown to sensitize cells to thiopurines.

Next to the clinically important role of Nudt15, its activity in hydrolyzing the 5′mRNA cap structure on RNA transcripts in vitro was shown (Song et.al. 2013). This suggested a possible role of Nud15 in 5′RNA cap turnover, in parallel to other members of the NUDIX family such as Dcp2, Nudt16, or Nudt3 (Wang et.al. 2002; van Dijk et.al. 2002; Grudzien-Nogalska et.al.^2016^; Grudzien-Nogalska and Kiledjian 2017). Nudt15 was shown also to hydrolyze the m^7^GDP mononucleotide—one of the decapping products in the 5′–3′ mRNA degradation pathway (Song et.al. 2013). To date, the available model of m^7^GDP elimination suggests a two-step process: first, m^7^GDP is converted to m^7^GTP by (possibly) nucleoside diphosphate kinase (NDK), and then m^7^GTP hydrolysis by DcpS enzyme to m^7^GMP which is further eliminated by the cytosolic nucleotidase cNIII (Taverniti et.al. 2015). In this report, we address Nudt15 activity toward m^7^GDP and its triphosphate counterpart (m^7^GTP) in more detail by enzyme kinetic and binding studies. Trimethylated guanosine mononucleotides (m_3_^2,2,7^GDP and m_3_^2,2,7^GTP) were analyzed also, as our preliminary data suggested Nudt15 activity toward this type of compounds and trimethylated cap analogs, present on several types of cellular RNA (e.g., snRNA and snoRNA (Warminski et.al. 2017).

## Results

### Comparison of the susceptibility of mononucleotide compounds to mNudt15-mediated hydrolysis

**Fig. 1:**
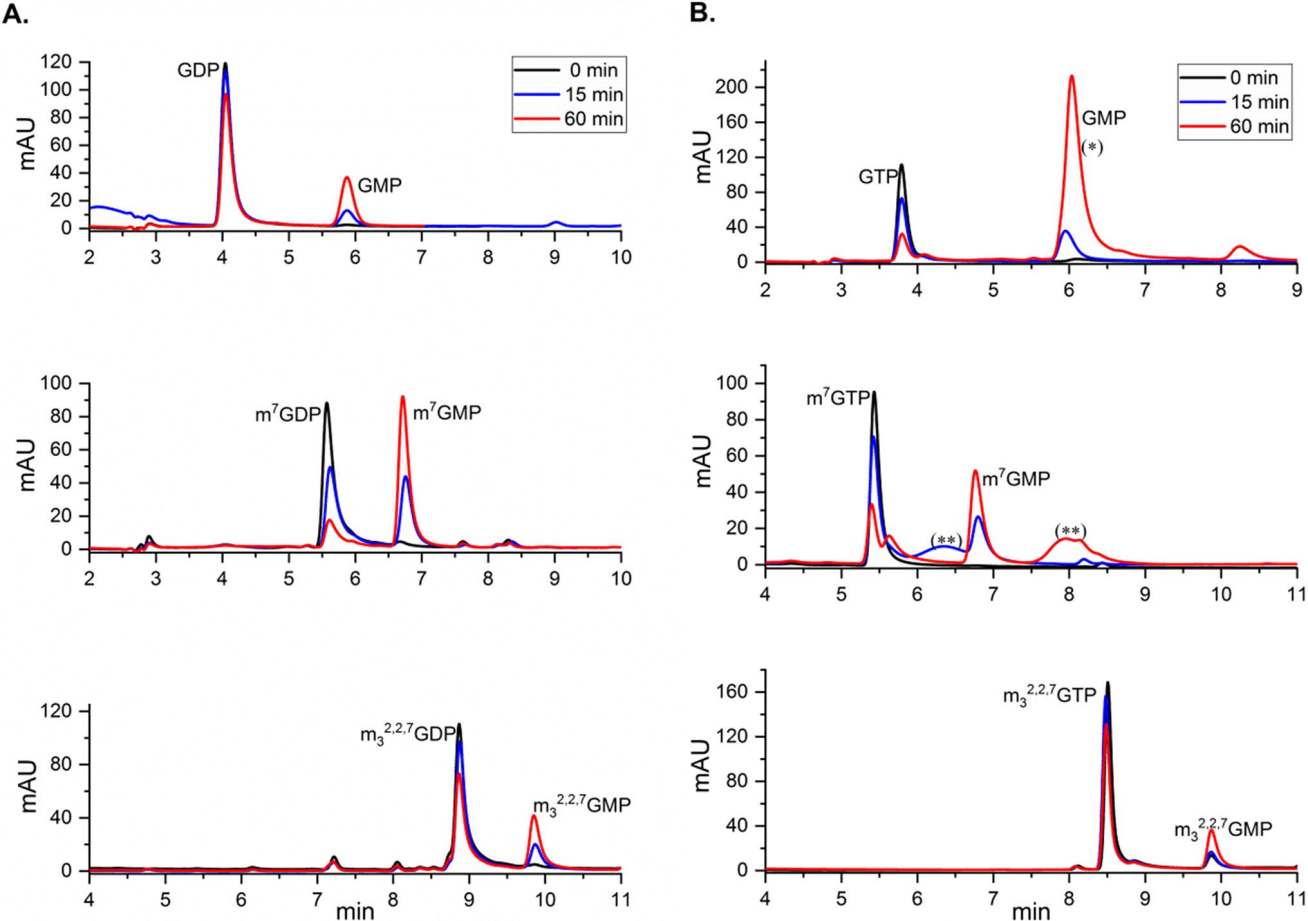
Representative RP-HPLC chromatograms of mNudt15-mediated hydrolysis of guanosine diphosphates (**A**) and guanosine triphosphates (**B**). The initial concentration of each indicated substrate was 25 μM, and chromatograms were recorded at the 260 nm wavelength. Analysis of reaction progress after 15 min and 1 h for the indicated compounds are shown. Chromatogram peaks of the analyzed substrate and the corresponding monophosphate product are indicated. (The higher area under the GMP peak after 60 min of reaction (*) could result due to an impurity co-eluting with GMP, as in the additional chromatogram recorded after 2 h of reaction, the area under the GMP peak is comparable to the initial substrate peak area, Supplementary Fig. 3A. Additional peaks (**) seen in the case of m^7^GTP chromatograms correspond to some impurities, as those are not present in the additional chromatograms for this compound, Supplementary Fig. 3B)

It was previously shown that m^7^GDP is hydrolyzed into m^7^GMP with murine Nudt15 under in vitro conditions (Song et.al. 2013), and our initial data showed that the trimethylated mononucleotide m_3_^2,2,7^GDP was also processed with mNudt15 (Supplementary Fig. 1). To investigate the susceptibility of these compounds to mNudt15 in more detail, enzymatic reactions with a wider set of monomethaled and trimethylated guanosine mononucleotides (m^7^GDP, m^7^GTP, m_3_^2,2,7^GDP and m_3_^2,2,7^GTP) were performed and the products of mNudt15-mediated cleavage were analyzed by RP-HPLC. Non-methylated counterparts (GDP and GTP) and known Nudt15 substrate (dGTP) were also included in the analysis. The canonical nucleotide dGTP was used in previous comparative enzymatic studies with the oxidized 8-oxo-dGTP and thionylated Nudt15 substrates (6-thio-dGTP and 6-thio-dGTP) (Carter et.al 2015; Valerie et.al. 2016), and the reproted kinetic parameters for dGTP are available for comparative analysis. As shown in the obtained RP-HPLC profiles, all investigated compounds were hydrolyzed with mNudt15 to different extents (Fig. 1). The methylated compounds analyzed (both in the diphosphate and in the triphosphate form) are hydrolyzed to the corresponding monophosphates. However, monomethylated m^7^GDP and m^7^GTP are more robustly processed (> 50% of substrate cleaved within 1 h) compared to their trimethylated counterparts under the same in vitro conditions used (Supplementary Table 1.). Also, it appears that m^7^GDP and m^7^GTP are hydrolyzed to a similar extent as GTP. Analysis of the reaction progress (Supplementary Fig. 2) revealed that the methyl moiety in m^7^GDP increases the hydrolysis rate value around 3× compared to GDP (3.59 μM/min vs 1.17 μM/min), and in the case of triphosphate compounds the presence of m^7^Guo in m^7^GTP decreases the hydrolysis rate about twofold compared to GTP (2.45 μM/min vs 5.16 μM/min) (Supplementary Table 2). In the case of trimethylated mononucleotides, the kinetic rate value for m_3_^2,2,7^GTP is one order of magnitude lower than that for GTP (0.56 μM/min vs 5.16 μM/min), but in the case of m_3_^2,2,7^GDP diphosphate is only two times lower than that for unmodified GDP (0.64 μM/min vs 1.17 μM/min). In the case of dGTP and GTP nucleotides, the ratio of hydrolysis rates reported here (dGTP/GTP ~ 10) is in a similar range to that presented by Valerie et.al. (2016).

**Fig. 2:**
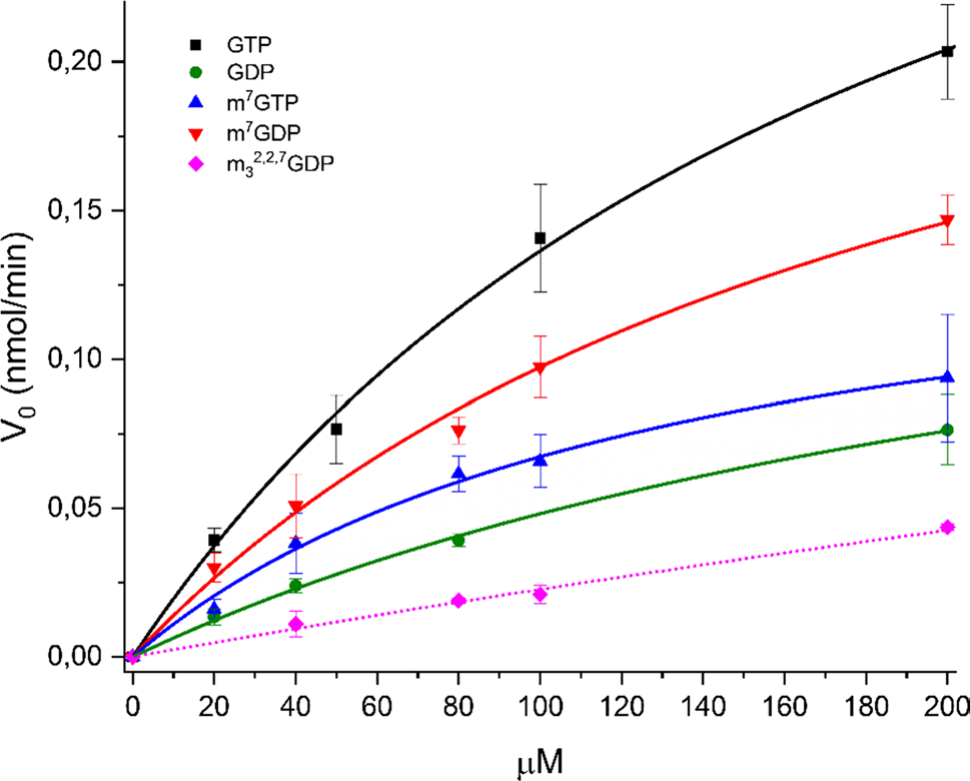
Kinetic curves of mNudt15-mediated hydrolysis of tested mononucleotides. Experimental data points correspond to the initial velocities (presented as nmol of the enzymatically released phosphate per minute) of three independent experiments (± SD), based on the colorimetric assay (as described in Methods). Fitted curves of Michaelis–Menten kinetic model to experimental data points are shown. Solid lines correspond to results obtained at 200 nM mNudt15 concentration, and dotted line to results for trimethylated mononucleotide obtained at 500 nM mNudt15 concentration (kinetic curve for dGTP was omitted for clarity)

To further characterize the substrate preferences of the analyzed mononucleotides for mNudt15, we performed a kinetic analysis using a wider range of compound concentrations (Fig. 2). The derived Michaelis–Menten kinetic constants are summarized in Table 1. As seen, the mNudt15 *K*_M_ value for dGTP is the same as reported earlier (79 μM vs. 75 μM, Cai et.al. 2003) and the *K*_M_ values for dGTP and GTP are in a similar range to those reported for human Nudt15 (79 μM vs. 43 μM in the case of dGTP, and 198 μM vs. 254 μM in the case of GTP, respectively) (Valerie et.al. 2016). m^7^GDP and m^7^GTP showed here similar (m^7^GDP *K*_M_ = 201 μM) or around 1.5-fold higher affinity (m^7^GTP *K*_M_ = 132 μM) in comparison to GTP (Table 1). In the case of trimethylated m_3_^2,2,7^GDP, fitting the Michaelis–Menten kinetic model to the experimental data did not yield reliable values for the kinetic constants. In comparison to the known physiological concentrations of dGTP and GDP (~ 5 μM and ~ 90 μM, respectively, Traut 1994), the *K*_M_ values reported here are much higher (79 μM and 276 μM). In the case of GTP, the *K*_M_ constant value is less than 50% of the physiological GTP concentration (~ 460 μM, Traut 1994). For the methylated counterparts of GDP/GTP, no data on their intracellular concentrations are available to date.

**Table 1:** Summary of mNudt15 kinetic parameters

Mononucleotide	K_M_ (μM)	Vmax (nmol/min)
dGTP	**79 ± 16**	**2.23 ± 0.25**
GTP	**198 ± 16**	**0.406 ± 0.017**
GDP	**276 ± 36**	**0.181 ± 0.016**
m^7^GTP	**132 ± 21**	**0.156 ± 0.014**
m^7^GDP	**201 ± 28**	**0.293 ± 0.025**
m_3_^2,2,7^GTP	**n.d**	**n.d**
m_3_^2,2,7^GDP	**n.d**	**n.d**

The* K*_M_ and Vmax Michaelis–Menten constants were determined from enzyme kinetic curves based on the colorimetric assay (Fig. 2). Presented values correspond to three independent experiments (± SD)

### Development of binding assay conditions for hydrolyzable Nudt15 compounds

To gain further insight into the interaction of the studied di- and triphosphate mononucleotide mNudt15substrates under non-hydrolyzable conditions, we first tested the binding conditions that do not support its enzymatic activity. The enzymatic activity of Nudt15 is dependent on the presence of magnesium or manganese divalent cations (Song et.al. 2013; Carter et.al. 2015). As other ions can also influence the activity of Nudix proteins (Peculis et.al. 2007; Lu et.al. 2011), the potential binding of Ca^2+^ and Zn^2+^ ions by Nudt15 and their effect on enzyme activity was analyzed. Ion binding experiments were performed using differential scanning fluorimetry method (DSF) (Niesen et.al. 2007). DSF measures the thermal unfolding of the analyzed protein in the presence of an environmentally sensitive fluorescent dye (e.g., SYPRO Orange) that binds to the exposed denaturation hydrophobic regions of protein, which changes its fluorescence intensity. Binding of a ligand to a protein usually increases its thermal stability (which results in shift of recorded DSF curves toward higher temperatures and increase in the protein melting temperature, Tm). All divalent metal ions tested here (Mn^2+^, Mg^2+^, Ca^2+^ and Zn^2+^) showed distinct thermal stabilization of mNudt15, indicating their interaction with the protein (Supplementary Fig. 4A). Among the tested ions, Zn^2+^ showed the highest stabilization of mNudt15 in low milimolar range (0.5–1 mM) in comparison to the ion-free form, and drastic destabilization at higher concentrations that resulted in flattening the DSF curve (Supplementary Fig. 4B). In the case of calcium ions, a gradual increase of the mNudt15 melting temperature (Tm) was seen in response to increasing concentrations of Ca^2+^ (up to 50 mM), with the maximal stabilization reached between 10 and 20 mM, similarly to magnesium ions (Supplementary Fig. 5). Based on these results, we performed initial analysis of ligand binding (dGTP) in the presence of zinc (1 mM) or calcium ions (10 mM). As shown in Supplementary Fig. 6, Ca^2+^ ions, but not Zn^2+^, enable effective binding of this hydrolyzable compound by mNudt15. RP-HPLC profiles of enzymatic reaction set up of dGTP with mNudt15 in the presence of calcium ions showed no reaction products at all (Supplementary Fig. 7), indicating that despite substrate binding mNudt15 is not active in the presence of Ca^2+^. A similar effect of calcium ions on the enzymatic activity of NUDIX proteins was reported for zfMTH1 (a close homolog of Nudt15) or Nudt12 (Jemth et.al. 2018; Lukaszewicz et.al. 2023). The resolved structure of zfMTH1 contains three calcium ions in the Nudix-box motif (PDB 5TON) and comparison of the binding calcium to zfMTH1 with the binding magnesium ions to human Nudt15 (PDB 5BON) has shown that two positions of Ca^2+^ and Mg^2+^ overlap and the third calcium ion binds to a different position in relation to the remaining two magnesium ions of Nudt15 (Jemth et.al. 2018). Such a distortion in a metal ion binding position in the case of Ca^2+^ combined with its larger ionic radius compared to Mg^2+^ (1.06 Å vs. 0.72 Å) and thus a change in the metal–ligand distance and a difference in the preferred coordination geometries (6 coordinated ligands in the case of Mg^2+^, and 7–8 in the case of Ca^2+^) (Peeraer et. al. 2004) could be involved in the catalytically inactive state of calcium-bound mNudt15.

### Analysis of the binding of guanosine mononucleotide derivatives to mNudt15 by DSF

Next, the binding experiments of dGTP, GDP, GTP, m^7^GDP, m^7^GTP, m_3_^2,2,7^GDP, and m_3_^2,2,7^GTP were performed by differential scanning fluorimetry in the presence of Ca^2+^ (instead of Mg^2+^), as we have showed that mNudt15 binds to a nucleotide ligand (dGTP) in the presence of calcium but is not catalytically active. The obtained results are summarized in Table 2. As seen, GDP, GTP, and m_3_^2,2,7^GTP have little effect on thermal stabilization of mNudt15 at the higher (500 μM) compound concentration (Δ*T*m ≤ 2 °C). m^7^GTP and m_3_^2,2,7^GDP stabilize mNudt15 to a similar extent as dGTP (Δ*T*m ~ 4 °C). Interestingly, m^7^GDP shows the highest thermal stabilization of mNudt15 (Δ*T*m = 7.4 °C) which is around 2× higher value compared to dGTP at the same concentration used (Table 2).

**Table 2:** Tm values (°C) of mNudt15 obtained at 200 μM and 500 μM concentration of the tested mononucleotide

	Tm (°C) (at 200 μM ligand conc.)	*T*m (°C) (at 500 μM ligand conc.)	Δ*T*m (°C)
dGTP	59.5 ± 0.9	61.1 ± 1.7	4.2
GTP	58 ± 0.4	58.8 ± 0.4	1.9
GDP	57 ± 0.4	58.5 ± 0.7	1.6
m^7^GTP	59. ± 0.4	60.8 ± 0.4	3.9
m^7^GDP	61.8 ± 0.6	64.2 ± 0.4	7.3
m_3_^2,2,7^GTP	59 ± 0.4	59 ± 0.4	2.1
m_3_^2,2,7^GDP	60.3 ± 0.4	61.3 ± 0.4	4.4

The Δ*T*m value corresponds to the difference between the *T*m value obtained at 500 μM compound concentration and Tm value of the mNudt15 ligand-free form (*T*m = 56.9 ± 0.5 °C). Tm values correspond to three independent experiments (± SD) (or ten in the case of ligand-free form of mNudt15).

**Fig. 3:**
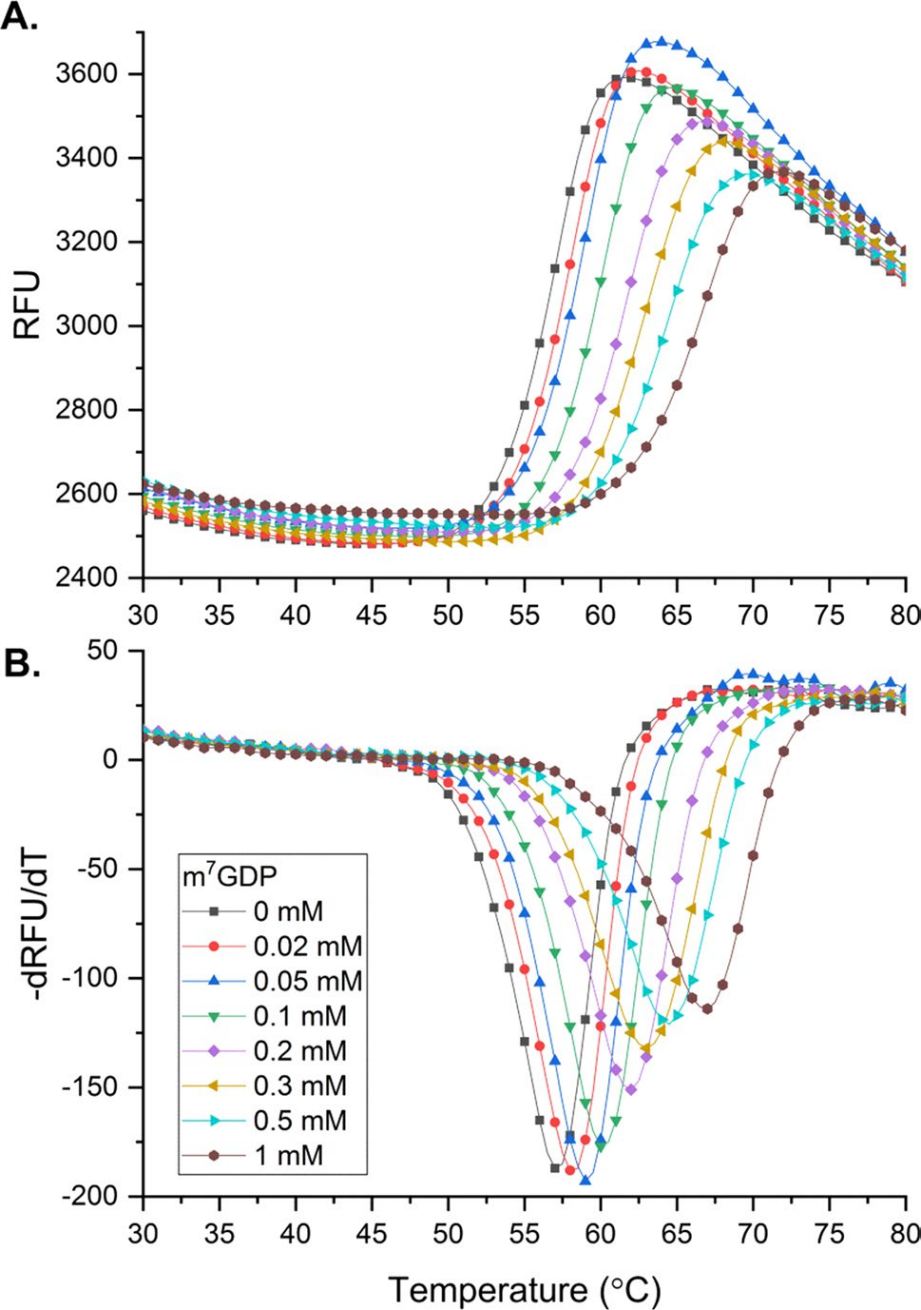
Thermal stabilization of mNudt15 over increasing concentrations of m^7^GDP. (**A**) Representative DSF melting profiles of mNudt15, RFU—relative fluorescence units. (**B**) Curves of the first negative derivative of the corresponding melting curves

**Fig. 4:**
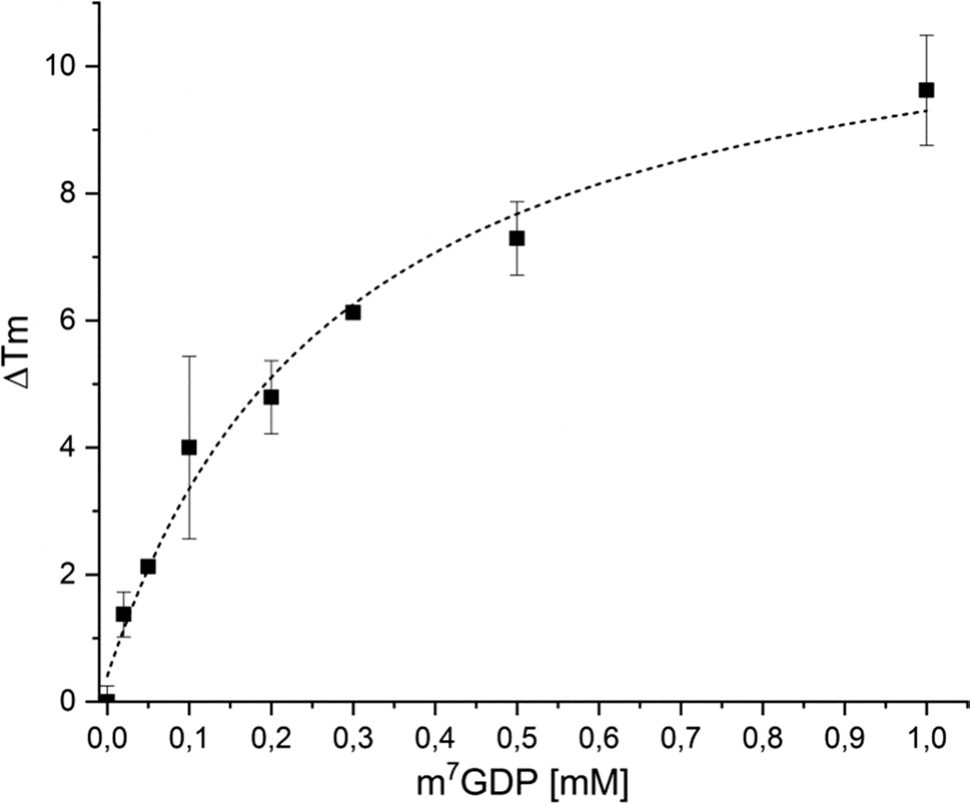
Thermal stabilization of mNudt15 as a function of m^7^GDP concentration. ΔTm values data points correspond to of three independent DSF experiments (± SD) and are plotted versus the corresponding concentration of the analyzed compound. Apparent affinity (*app*.K) was calculated as described by Vivoli et.al. (2014), by fitting to the experimental data to the single site ligand binding model, dashed line (Origin Pro)

In the case of m^7^GDP, the DSF experiment was performed for a wider compound concentration from 0 to 1000 μM (Fig. 3). The obtained data revealed a gradual increase in the thermal stabilization of mNudt15 upon m^7^GDP binding (shift of the recorded DSF melting curves toward higher temperatures, Fig. 3A). Based on the change in Tm in response to the increasing concentration of m^7^GDP (Fig. 4), its apparent binding affinity to mNudt15 was estimated with *app*. *K* value of 285 ± 71 μM.

## Discussion

Since Nudt15 has been shown to play a crucial role in the metabolism of thiopurine drugs, numerous genotyping studies and characterization of Nudt15 variants were undertaken (Suiter et.al. 2020; Pratt et.al. 2022) and compounds specifically targeting Nudt15 are being developed (Zhang et.al. 2020; Rehling et.al. 2021). As deletion of Nudt15 does not affect the viability or result in any histological abnormalities (based on mouse model organism, Nishii et.al. 2018), the physiological function of Nudt15 is still unclear. Analysis of the substrate specificity of Nudt15 showed moderate activity in vitro toward several types of nucleotide compounds, including canonical and modified (d)NTPs, 8-oxo-dGTP, 8-oxo-dGTP, 5-me-CTP, dGTP, and GTP (Takagi et.al. 2012; Carreras-Puigvert et.al. 2017), and higher activity toward 6-thio-(d)GTP (Carter et.al. 2015; Valerie et.al. 2016) or ganciclovir triphosphate (Zhang et.al. 2021). Although enzymatic activity has been shown for yet another Nudt15 substrate—m^7^GDP (Song et.al. 2013), no further detailed studies have been reported to date. Since m^7^GDP is one of the products of 5′-end decapping of mRNA in the process of mRNA degradation, it is an open question whether Nudt15 may contribute in this process by subsequently metabolizing this cap-derived methylated mononucleotide.

The data obtained here show that in addition to m^7^GDP, m^7^GTP is also effectively hydrolyzed by Nudt15 to a similar extent as GTP (more than 60% of the initial substrate concentration is hydrolyzed in each case under the same experimental conditions), but with a slightly reduced rate of hydrolysis (about 1.4 times for m^7^GDP and about 2.1 times for m^7^GTP). A similar difference is seen in the calculated Vmax values for these compounds. Thus, the presence of the methyl moiety seems to interfere here with the enzymatic hydrolysis compared to GTP. However, in the case of diphosphate compounds (GDP vs. m^7^GDP), the positive effect of the presence of the 7-methyl moiety is seen as it results in a threefold increase in the hydrolysis rate of m^7^GDP with Nudt15. Interestingly, the calculated K_M_ values for m^7^GDP and GTP do not differ, whereas the K_M_ for m^7^GTP is 1.5 times lower (compared to GTP and m^7^GDP). Comparison of the hydrolysis rate values of m^7^GDP and m^7^GTP with that calculated for dGTP shows approximately 95% reduction in the catalytic rate. This, in turn, implies that the activity of Nudt15 toward these monomethylated compounds in in vitro studies is unlikely to be relevant in vivo and represents yet another example of a substrate toward which Nudt15 has moderate enzymatic activity. In the case of the trimethylated nucleotides, m_3_^2,2,7^GDP and m_3_^2,2,7^GTP, although hydrolysis products are readily detectable after 1 h of incubation with Nudt15, their hydrolysis rates are four to five times lower compared to their monomethylated counterparts. Therefore, they are not preferred Nudt15 substrates, as shown here in vitro. However, it should be noted that the activity of the murine Nudt15, used in the present studies, may differ from that of human Nudt15, despite a very high amino acid sequence identity (Rehling et.al. 2021). Such a difference was observed in the case of one of the Nudt15 inhibitors, TH7755 (Rehling et.al. 2021). Nerveless, as mouse models are used in testing potential therapeutics prior to human clinical trials, mNudt15 remains important area of studies.

Finally, despite the results obtained in Nudt15 enzymatic activity experiments, intriguing data are seen in a ligand binding studies. Here, the methylated diphosphate mononucleotides (both mono- and trimethylated) show a distinct stabilization of Nudt15, in the same (for m_3_^2,2,7^GDP) or 2× higher (for m^7^GDP) range compared to dGTP. Interestingly, the available thermal stabilization data for the initially identified inhibitory compound of hNudt15 (TH884, with IC_50_ = 12.5 μM) shows an increase in the melting temperature by ~ 3 °C at 50 μM concentration (Zhang et.al. 2020). This is similar to the Δ*T*m value (~ 2 °C) reported here for m^7^GDP at the same 50 μM concentration. As m^7^GDP appears to be effectively bound by Nudt15 and trimethylated m_3_^2,2,7^GDP is poorly hydrolyzed despite a clear stabilization of Nudt15 (in the same range as dGTP) in the DSF approach, it would be interesting to elucidate in future the molecular features of their interaction with Nudt15. Such data could be useful in the design of small molecule inhibitors of Nudt15.

## Materials and methods

### Purification of murine Nudt15 (mNudt15)

Recombinant mNudt15 was purified as N-His-tagged protein from *E. coli* Rosetta 2 (DE3) transformed with the pET28a-Nudt15vector (Song et.al. 2013). Shortly, O/N bacterial culture (LB medium with 50 μg/mL kanamycin) was diluted 1:100 into 1 L of LB with kanamycin and incubated at 37 °C with shaking until OD600 reached ~ 0.5. The culture was then induced with 0.2 mM IPTG and incubated O/N at 20 °C with shaking. Bacterial cells were harvested by centrifugation (7700 rpm, 8 °C), washed in 1×PBS, and collected again (6000 rpm, 8 °C).

The obtained cell pellet was resuspended in lysis buffer (20 mM HEPES–KOH pH 8.0, 300 mM NaCl, 300 mM urea, 10 mM imidazole, 10% glycerol, 1% Triton X-100) supplemented with lysozyme, incubated on ice for 30 min, and disrupted by sonication. After centrifugation (20,000×*g* for 30 min), the supernatant was filtered (syringe filter, 0.45 μm) and incubated at 4 °C for 1 h with gentle stirring in the presence of 2.5 mL HIS-Select Nickel Affinity Gel (Sigma) equilibrated with binding buffer (20 mM HEPES–KOH, pH 8.0, 300 mM NaCl, 10 mM imidazole, 10% glycerol). The affinity resin with bound protein was loaded into a polypropylene column) and washed with 20 mL of wash buffer (20 mM Tris–HCl, 300 mM NaCl). The His-tagged protein mNudt15 was subsequently eluted with increasing concentrations of imidazole (from 20 to 300 mM) in the wash buffer (20 mM Tris–HCl, 300 mM NaCl). Obtained mNudt15 fractions were finally purified by gel filtration on a Superdex200 Increase column in elution buffer (50 mM HEPES pH 8.0, 150 mM KCl) using NGC Chromatography FPLC system (Bio-Rad). For storage at ‒80 °C, protein samples were supplemented with 10% glycerol and 1 mM DTT (final concentrations) and flash-frozen in liquid nitrogen. Fractions of mNudt15 after each step of the purification procedure were analyzed by SDS-PAGE electrophoresis in 12% polyacrylamide gels (Supplementary Fig. 8).

### Enzymatic assays

mNud15-mediated hydrolysis of mononucleotide compounds (25 μM) was performed in the assay buffer (50 mM HEPES, 100 mM KCl, 10 mM MgCl_2_, 2 mM DTT, pH 8.0) with 200 nM final enzyme concentration, at 30 °C for the indicated time periods. The reaction was stopped by heating the mixture for 5 min at 95 °C. The reaction products were analyzed by HPLC (Agilent 1200 series) equipped with a reverse-phase Supelcosil LC-18-T column and a UV/VIS detector. The reaction mixture was loaded directly into the HPLC column and elution was performed at 20 °C with a linear gradient of methanol in 0.1 M KH_2_PO_4_ (from 0 to 25% for unmethylated compounds, from 0 to 25% for monomethylated compounds, and from 0 to 40% for trimethylated compounds) over 15 min at a flow rate of 1.0 mL/min. Changes in the absorbance at 260 nm were continuously monitored during the analysis.

For hydrolysis rates determination experiments, the concentration of the investigated compounds was 25 μM in the reaction mixture and, to facilitate the detection of reaction products over the initial reaction period, the enzyme concentration was adjusted according to the type of substrate (500 nM for m_3_^2,2,7^GDP and m_3_^2,2,7^GTP, 300 nM for GDP, 200 nM for GTP, m^7^GDP and m^7^GTP, 10 nM for dGTP). To analyze the reaction progress, 200 μL samples of the reaction mixture were withdrawn at specified time points (0, 2, 4, 6, 9 and 15 min) of reaction progress and analyzed by RP-HPLC. The extent of hydrolysis was determined using the area under the chromatographic peaks of the respective compounds. The initial velocity values were calculated from three independent experiments.

Kinetic analysis of the mNudt15 enzyme was performed with the tested mononucleotides at concentrations ranging from 0 to 200 μM. In the case of diphosphate mononucleotides, cleaved to the corresponding monophosphate and inorganic phosphate, the reaction progress was monitored by colorimetric detection of the reaction-formed inorganic phosphate with BIOMOL Green reagent (Enzo Life Sciences). In the case of triphosphate mononucleotides, the reaction-formed pyrophosphate was converted by yeast inorganic pyrophosphatase (NEB) to inorganic phosphate that was subsequently detected with BIOMOL Green reagent (Zhang et.al. 2020). Enzymatic reactions were performed in the assay buffer (50 mM HEPES, 100 mM KCl, 10 mM MgCl_2_, 2 mM DTT, pH 8.0) at 30 °C with 200 nM final enzyme concentration (or 500 nM in the case of trimethylated compounds). Where appropriate, yeast inorganic pyrophosphatase was added to the reaction mixture at a final concentration 0.2U/ml. Reactions were stopped at the indicated time periods by removing aliquots of the reaction mixture and adding ice-cooled EDTA to 50 mM final concentration. After that, BIOMOL Green reagent was added, and after 30 min of incubation at room temperature the absorbance was measured at 620 nm with microplate reader (H1 Synergy, Biotek). An inorganic phosphate standard curve ranging from 0 to 2 nmol was performed for each set of assay data. Determination of thekinetic parameters for mNudt15 was performed by calculating the initial velocity rates by linear regression and fitting the obtained initial rate data to the Michaelis–Menten kinetic model (Origin Pro. software).

### Analysis of binding of mononucleotide substrates to mNudt15 by DSF

The thermal stability of hNudt15 was analyzed using DSF (Niessen et.al. 2007). The assay sample (20 μl) contained 4 × SYPRO Orange (Sigma Aldrich) and 4 μM mNud15 (final concentrations) in 50 mM HEPES, 100 mM KCl, 20 mM CaCl_2_, 2 mM DTT (pH 8.0). Tested compounds were prepared as10× concentrated stocks (in relation to final concentrations); 2 μl was added to the assay sample, and the reaction mixture was incubated for 10 min on ice. A CFX96 Real-Time PCR (Bio-Rad) was used to increase the temperature from 25 °C to 95 °C in 0.5 °C increments (1 °C/min), and fluorescence intensity (FRET channel, with excitation in the 450–490 nm range, and emission in the 560–580 nm range) was measured at each step. The melting temperature (Tm) was calculated using the CFX Manager Software (Bio-Rad) as the minimum of the first negative derivative of the DSF melting curves.

### Mononucleotide compounds used in this study

GTP and dGTP were purchased from Fermentas. m^7^GDP, m^7^GTP, m_3_^2,2,7^GDP and m_3_^2,2,7^GTP were synthesized according to the methodology described previously (Jankowska et.al. 1993; Niedzwiecka et.al. 2007).

### Supplementary Information

Below is the link to the electronic supplementary material.

Supplementary file1 (PDF 1542 KB)

## Data Availability

Data are available upon request.
