# Supplementary material for: Mammalian Nudt15 hydrolytic and binding activity on methylated guanosine mononucleotides
Source: Eur Biophys J. 2023 Aug 29;52(6-7):487–95. doi: 10.1007/s00249-023-01678-5 (PMC10618335; doi:10.1007/s00249-023-01678-5)
Supplement: Supplementary file 1 — Supplementary file1 (PDF 1542 KB) [file 249_2023_1678_MOESM1_ESM.pdf]

Supplementary Information

Mammalian Nudt15 hydrolytic and binding activity on methylated guanosine mononucleotides

Maciej Lukaszewicz[1*] , Aleksandra Ferenc-Mrozek[1], Julia Kokosza[1], Anna Stefaniuk[1], Janusz Stepinski[1], Elzbieta Bojarska[1], Edward Darzynkiewicz[1,2]

[1] Department of Biophysics, Faculty of Physics, University of Warsaw, Pasteura 5, 02-093 Warsaw, Poland

[2] Centre of New Technologies, University of Warsaw, Banacha 2c, 02-097 Warsaw, Poland

Corresponding author[*]: Maciej Lukaszewicz, Department of Biophysics, Faculty of Physics, University of Warsaw, Pasteura 5, 02-093 Warsaw, Poland
Tel: +48 22 5532353, e-mail: Maciej.Lukaszewicz@fuw.edu.pl

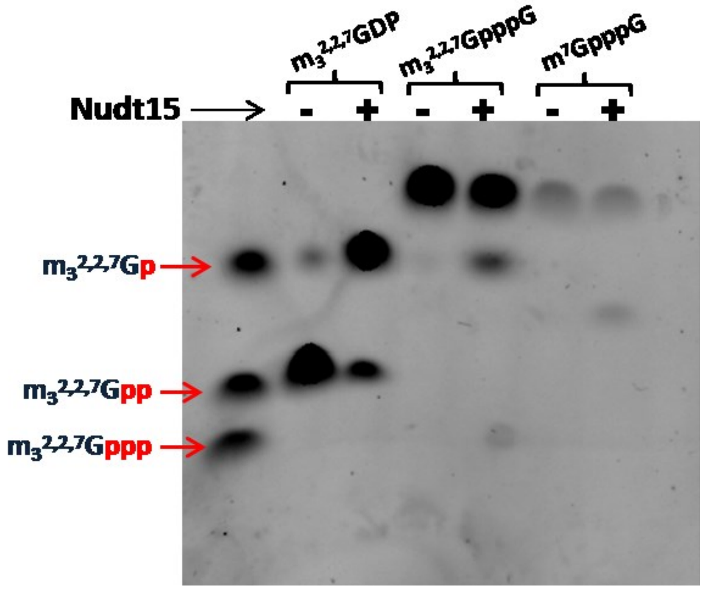

Supplementary Figure 1A. Enzymatic activity of mNudt15 towards $m_3^{2,2,7}$GDP, $m_3^{2,2,7}$GpppG and m^7GpppG. Reaction products were resolved electrophoreticaly on 20% polyacrylamide denaturing gel with 7M urea in 1x TBE buffer, at constant voltage 400V for 3 hours. After that the gel was placed directly onto UV lamp (302 nm, ChemiDoc Biorad) and substrates and products were visualized under transmitted UV light (absorption spectra of example trimethylated and monomethylated compounds shown in Supplementary Figure 1B). Migration of standard mononucleotides are indicated with red arrows. 300 μM of indicated substrate was incubated with 2 μM mNudt15 at 30°C for 1 hour in reaction buffer (10mM Tris-Cl, pH 7.5, 100mM KCl, 2mM $MgCl_2$, 2mM DTT, 0.5mM $MnCl_2$, 40U/ml RNase inhibitor, Song *et.al.* 2013). Reactions were stopped by adding equal volume of formazol, and loaded onto prepared gel. As it is seen, under reaction conditions used, $m_3^{2,2,7}$GDP mononucleotide is hydrolyzed into its monophosphate form, whereas dinucleotide 5'end RNA cap analogs ($m_3^{2,2,7}$GpppG and m^7GpppG) are poorly processed with mNudt15.

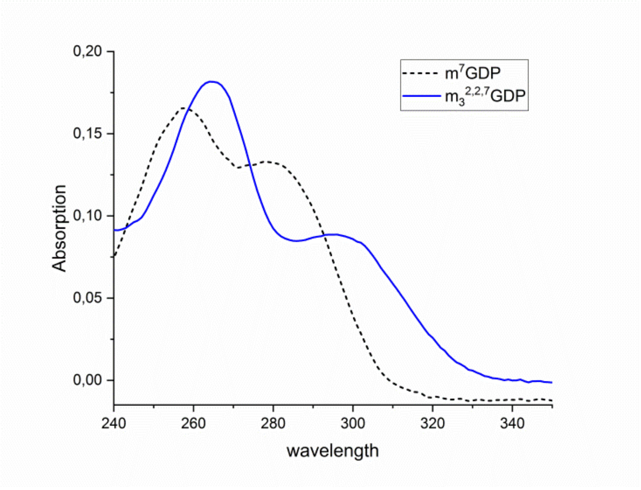

Supplementary Figure 1B. Absorption spectra of 20μM m^7GDP and $m_3^{2,2,7}$GDP (in 0.1M Phosphate buffer, pH 7.0).

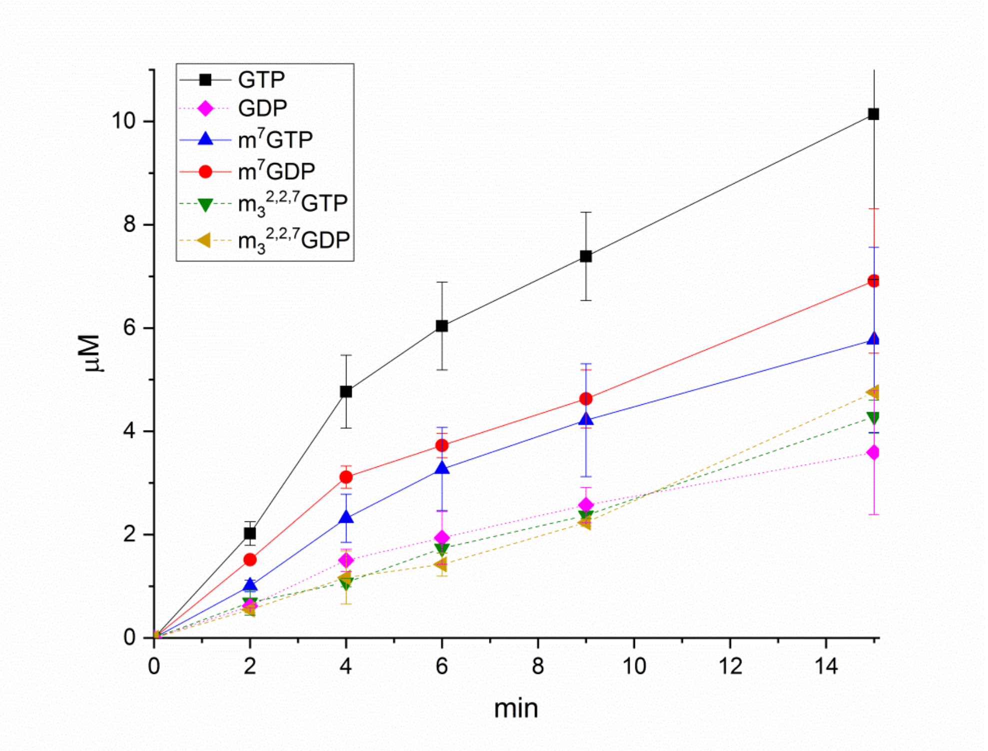

Supplementary Figure 2. Reaction progress of mNudt15-mediated hydrolysis of indicated guanosine mononucleotides based on HPLC assay. 25µM of each substrate (initial concentration) was subject to enzymatic hydrolysis with 200 nM mNudt15 (for GTP, m^7GTP and m^7GDP), 300 nM mNudt15 for GDP, or 500 nM mNudt15 (for $m_3^{2,2,7}$GTP and $m_3^{2,2,7}$GDP), as described in Materials and Methods. Amount of hydrolysed substrate (in µM) over the reaction time (min) is shown in the graph. Data points (±SD) correspond to three independent experiments.

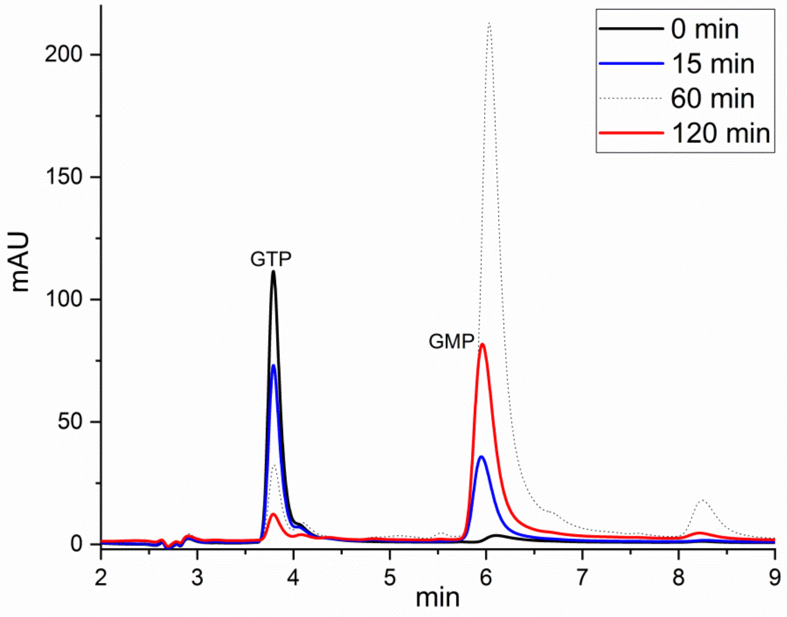

Supplementary Figure 3A. RP-HPLC analysis of mNudt15-mediated hydrolysis of GTP shown in **Figure 1** with additional chromatogram recorded after 2 hours of reaction.

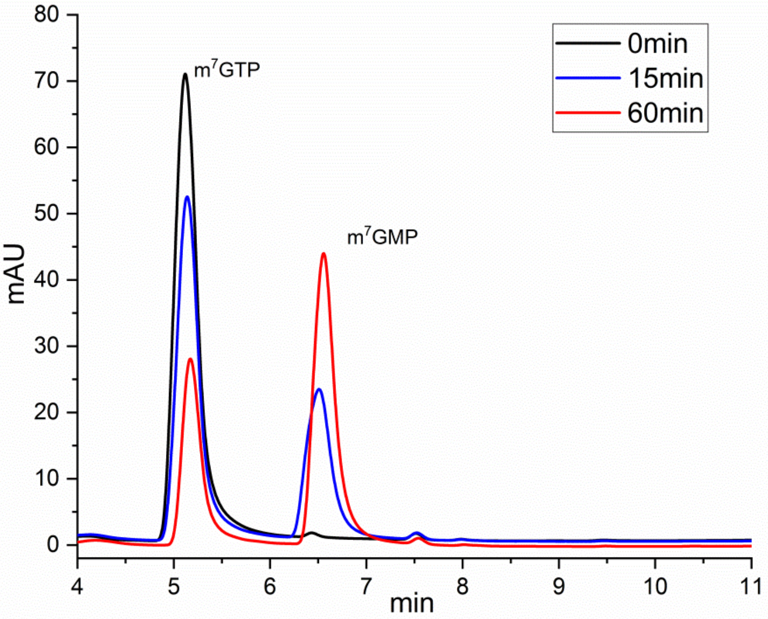

Supplementary Figure 3B. RP-HPLC analysis of mNudt15-mediated hydrolysis of m^7GTP (as shown in **Figure 1**).

A. **B.**

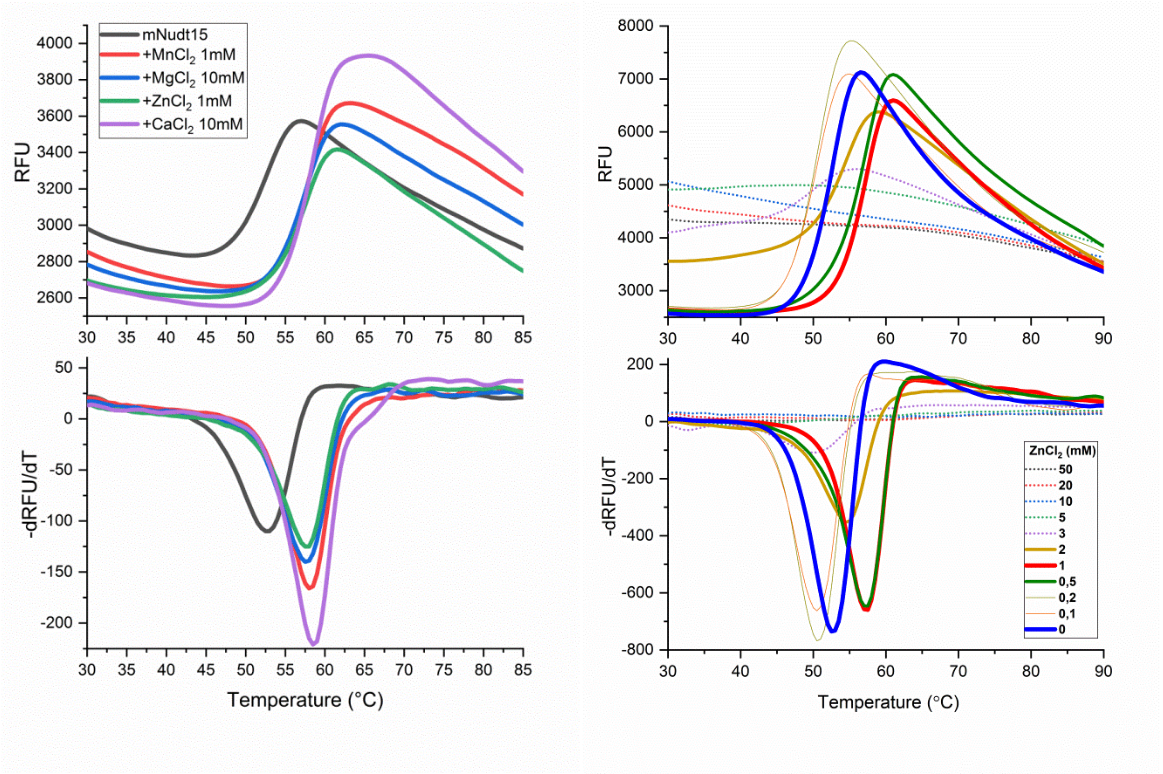

Supplementary Figure 4. Thermal stability of mNudt15 in the presence of indicated divalent ions analyzed by Differntial Scanning Fluorimetry (DSF).

(**A)** Representative DSF melting curves (upper panel) and corresponding curves of the first negative derivative of melting curves (lower panel) are shown. DSF experiments were performed with 4 µM of mNudt15 and 4x SYPRO Orange (final concentrations), in 50 mM HEPES/KOH buffer with 100 mM KCl and 2 mM DTT (pH 8.0), in the presence of 0.05 to 50 mM concentration of indicated salts. Melting curves obtained for indicated salt concentration that gave the highest observed positive shift in melting temperature (Tm) are shown in figure.

(**B)** Thermal stability of mNudt15 in the presence of increasing concentrations of $ZnCl_2$ (0.1 to 50 mM) in the DSF experimental conditions described above. The bold solid lines correspond to DSF curves obtained for mNudt15 in the absence of ions (blue line) and at 0.5, 1, and 2 mM $ZnCl_2$ (green, red and orange line, respectively). Dashed lines correspond to DSF melting curves (upper panel) recorded at 3 mM and higher concentrations (5-50mM) of $ZnCl_2$.

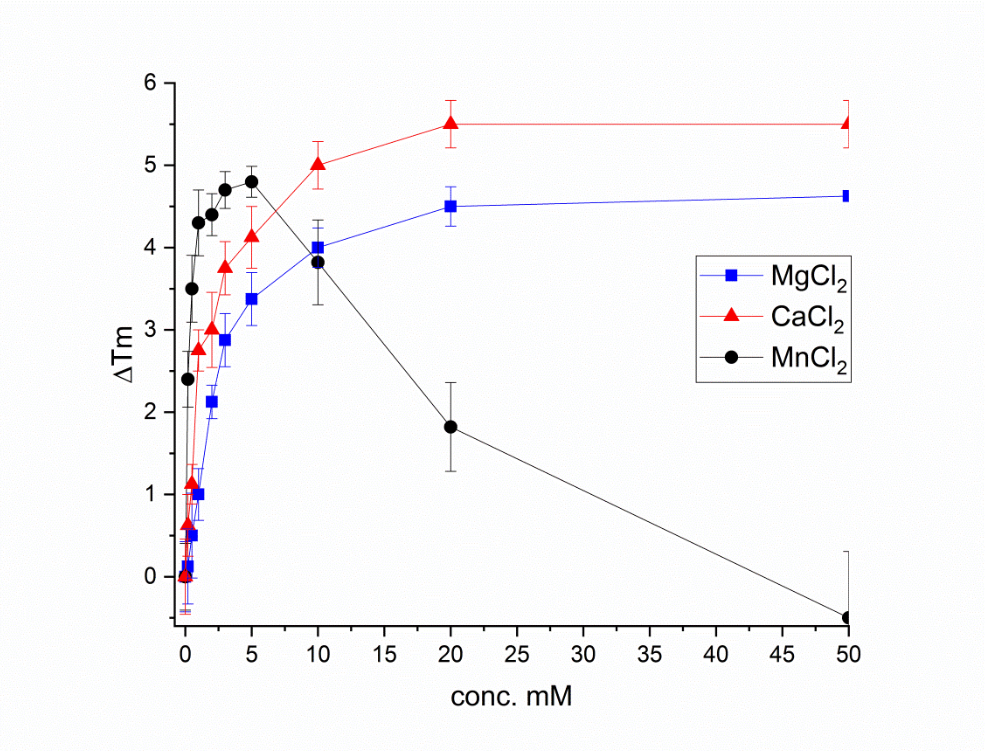

Supplementary Figure 5. Comparison of thermal stabilization of mNudt15 in the presence of increasing concentrations of $MgCl_2$, $CaCl_2$ and $MnCl_2$ (in DSF experimental conditions shown in Supplementary Figure 4A). ΔTm values correspond to the difference between Tm value obtained for mNudt15 in ion-free conditions and Tm value obtained at the particular concentration of tested salt.

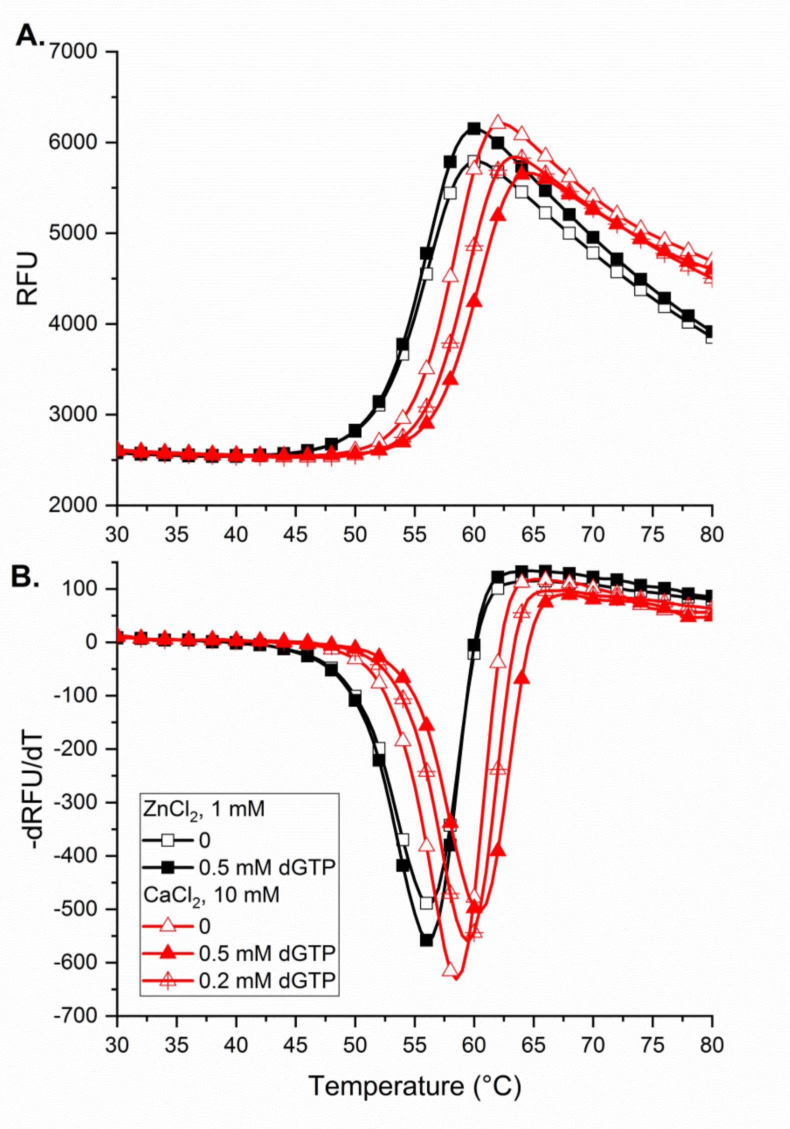

Supplementary Figure 6. Analysis of dGTP binding by mNudt15 in the presence of 1mM $ZnCl_2$ or 10mM $CaCl_2$. As shown, $CaCl_2$ at concentration that effectively stabilizes mNudt15 (Supplementary Fig.4A) enables binding of dGTP – hydrolysable substrate for mNudt15 *in vitro* (Valerie *et.al.* 2016). In contrast, $ZnCl_2$ at 1 mM concentration (that effectively stabilizes mNudt15, Supplementary Fig.4) do not show binding of dGTP.

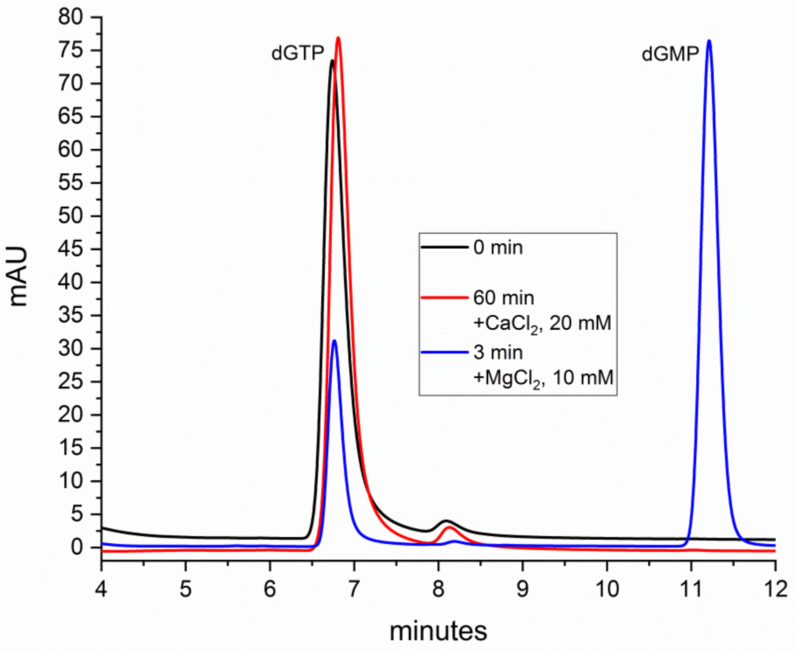

Supplementary Figure 7. Comparison of enzymatic activity of mNudt15 in the presence of Ca^{2+} or Mg^{2+} ions. Reaction mixtures of 25µM dGTP with 200 nM of mNudt15 and 20mM $CaCl_2$ after 0 min and after 60 min of incubation at 30°C were separated by RP-HPLC. As it is seen, no reaction product is detected at indicated time points in these experimental conditions (black and red line). In contrast, in the presence of 10mM $MgCl_2$ dGTP (25µM) is effectively hydrolyzed just after 3 min of incubation with 50 nM of mNudt15.

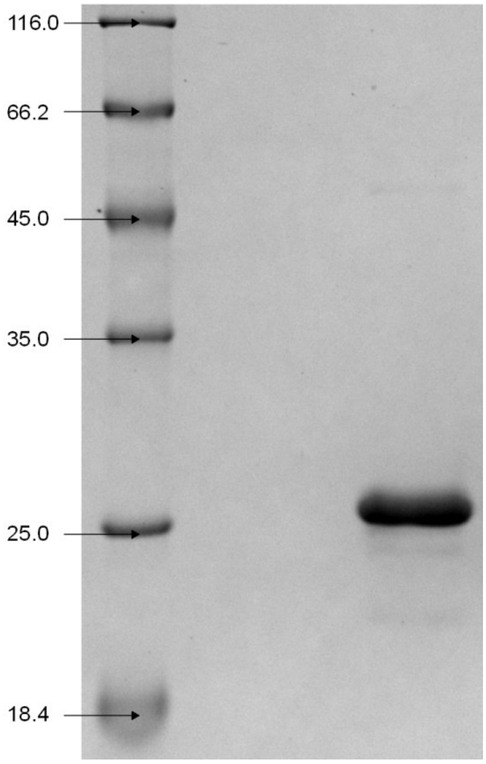

Supplementary Figure 8. Electrophoretic analysis of purified N-terminally His-tagged murine mNudt15 (in 12% polyacrylamide SDS-PAGE gel). Protein marker bands (left line) are marked with corresponding molecular weight values (in kDa).

Supplementary Table 1. Comparison of susceptibility of guanosine mononucleotides to enzymatic hydrolysis with murine Nudt15. Substrates and products were analyzed by RP-HPLC as described in Materials and Methods. Indicated percent values correspond to the fraction of hydrolyzed substrate (initial concentration 25µM) with 200 nM of mNudt15 after 1 hour of reaction (calculated as ratio of the HPLC substrate peak area after 60 min to the peak area at t=0min)

	fraction of substrate hydrolyzed after 60 min of reaction
GTP	67%
GDP	23%
m^7GTP	66%
m^7GDP	79%
$m_3^{2,2,7}$GTP	21%
$m_3^{2,2,7}$GDP	31%

Supplementary Table 2. Hydrolysis rates of mononucleotide compounds catalyzed by murine Nudt15 based on HPLC assay (Supplemenary Figure 2). Hydrolysis rates correspond to hydrolyzed substrate (μM) per minute per enzyme (μM) (1/min), for 25 μM initial substrate concentration. Data of three independent experiments (±SD) are presented

mononucleotide	Hydrolysis rate (1/min)
dGTP	**55.2 ± 16.8**
GTP	**5.16 ± 1.32**
GDP	**1.17 ± 0.17**
m^7GTP	**2.45 ± 0.77**
m^7GDP	**3.59 ± 0.57**
$m_3^{2,2,7}$GTP	**0.56 ± 0.05**
$m_3^{2,2,7}$GDP	**0.64 ± 0.01**

Supplementary references.

Song MG1, Bail S, Kiledjian M. Multiple Nudix family proteins possess mRNA decapping activity. RNA. **2013**, 19(3):390-9. doi: 10.1261/rna.037309.112.

Valerie NC, Hagenkort A, Page BD, Masuyer G, Rehling D, Carter M, Bevc L, Herr P, Homan E, Sheppard NG, Stenmark P, Jemth AS, Helleday T. NUDT15 Hydrolyzes 6-Thio-DeoxyGTP to Mediate the Anticancer Efficacy of 6-Thioguanine. Cancer Res. **2016,** 76(18):5501-11.
